# Preoperative facet joint arthropathy does not impact long-term clinical outcomes after lumbar-stability-preserving decompression and dynesys fixation

**DOI:** 10.1038/s41598-021-90967-0

**Published:** 2021-05-28

**Authors:** Po-Hsin Chou, Hsi-Hsien Lin, Yu-Cheng Yao, Shih-Tien Wang, Ming-Chau Chang, Chien-Lin Liu

**Affiliations:** 1 School of Medicine, National Yang Ming Chiao Tung University, No.155, Sec.2, Linong Street, Beitou District, Taipei, 112 Taiwan, ROC; 2 Department of Orthopedics and Traumatology, Taipei Veterans General Hospital, 18F, 201, Section 2, Shipai Road, Beitou District, Taipei, 112 Taiwan, ROC

**Keywords:** Diseases, Orthopaedics

## Abstract

To evaluate the impact of the preoperative severity of facet joint arthropathy on long-term functional outcomes and spinopelvic parameters in patients undergoing lumbar-stability-preserving decompression and Dynesys fixation. In this retrospective study, 88 patients undergoing combined surgery at our hospital from 2008 to 2015 were included. The patients were divided into two groups, the less and more than mean degeneration groups, based on preoperative facet joint arthropathy of the index level(s). The clinical outcomes were the Visual Analogue Scale (VAS) score, the Oswestry Disability Index (ODI) score and spinopelvic parameters. The mean follow-up durations for the less and more than mean degeneration groups were 84.83 ± 27.58 and 92.83 ± 20.45 months, respectively. The combined surgery significantly improved VAS and ODI scores, and increased sacral slope (SS) regardless of preoperative arthropathy severity. In addition, facet joint arthropathy at adjacent levels continued to worsen after surgery in both arthropathy severity groups. Preoperative facet joint arthropathy did not influence most long-term clinical outcomes in patients undergoing lumbar-stability-preserving decompression and Dynesys fixation. This combined surgery may be suitable for patients with facet joint arthropathy regardless of disease severity.

## Introduction

Facet joints, synovial joints between the superior and inferior articular processes of two adjacent vertebrae, contain hyaline cartilage surfaces and are innervated by the medial branch nerves^1^. Facet joints partially support axial compressive forces as well as rotational and shear forces acting on the lumbar spine, thereby playing a key role in load transmission between vertebrae^2^. Facet joint arthropathy, also called as facet joint osteoarthrosis, is degenerative arthritis involving cartilage degradation, facet joint space reduction, and osteophyte formation, which is often associated with chronic low back pain, sciatica, and neurogenic claudication^1,3,4^. A community-based population study revealed that 89% of adults aged 65 and over had moderate to severe lumbar facet joint arthropathy, and found that the prevalence and severity of lumbar facet joint arthropathy increase with age^5^. A cross-sectional study observed that women aged 50 and older were at a higher risk of lumbar facet joint arthropathy than male counterparts^6^. In addition, several other risk factors for lumbar facet joint arthropathy were proposed, such as higher body mass index (BMI), sagittal orientation of the facet joints, poor spinal extensors, and higher values of pelvic incidence^3,6,7^.

Chronic low back pain along with sciatica can be conservatively managed with physical therapy and pain medication^1^. For patients failing conservative management, lumbar radiofrequency ablation of the medial branch nerves may be performed^8^. Such nerve ablation causes sensory or sympathetic denervation, thereby ameliorating low back pain; however, recurrence of lower back pain may happen because of axonal regeneration^1,9^. On the other hand, lumbar facetectomy may be performed to treat facet arthropathy, osteophytes, or large synovial cysts with lateral recess and foraminal stenosis^1^. In addition, two surgical procedures, transforaminal lumbar interbody fusion (TLIF) and the non-fusion Dynesys dynamic stabilization, are often performed to treat lumbar facet joint syndrome with lateral recess stenosis caused by hypertrophied facet joints^10,11^. Several lines of evidence indicated that compared to TLIF, Dynesys dynamic stabilization achieved better or equal improvements in functional outcomes based on the Visual Analog Scale (VAS) and/or Oswestry Disability Index (ODI) scores^12–14^.

The Dynesys system, a commercial pedicle screw-based dynamic stabilization system, was designed to restrict segmental motion and to reduce intradiscal pressure and facet joint forces, which can be utilized for single or multilevel lumbar stabilization^11,15^. On the other hand, the beneficial effect of stability-preserving decompression in spinal lateral recess and foraminal stenosis was suggested^16^. Hence, it is reasonable that Dynesys dynamic stabilization has commonly been combined with decompression in the treatment of lumbar degenerative diseases with good results^17,18^. We have applied the combination of lumbar-stability preserving decompression and Dynesys fixation to patients with facet joint arthropathy with lateral recess and/or foraminal stenosis for nearly two decades.

The lumbar facet is essential for load distribution and spine stability^19^. However, whether the combined surgical procedures are suitable for all patients with facet joint arthropathy regardless of preoperative disease severity is unclear. The aim of the present retrospective study was therefore to evaluate the long-term impact of preoperative severity of facet joint arthropathy on functional outcomes and spinopelvic alignment in patients undergoing lumbar-stability preserving decompression and Dynesys fixation. The anticipated results may help to clarify the surgical indication for lumbar-stability preserving decompression and Dynesys fixation.

## Materials and methods

### Patient selection

This retrospective study was approved by the Institutional Review Board (IRB) (No. 2017-10-008BC) of our Hospital, and the requirement for informed consent was waived by the same IRB because of the retrospective nature of this study. All procedures performed in the present study were in accordance with the ethical standards of the Human Research Protection Center and the Helsinkin Declaration.

Patients who underwent lumbar-stability-preserving decompression and Dynesys fixation at our Hospital from 2008 to 2015 were initially selected. The combined surgical procedures were applied to patients who had lumbar degenerative spondylolisthesis and stenosis, patients who had neurogenic claudication or sciatica, or patients with lumbar disorders who failed to respond to conservative treatments for at least 3 to 6 months. Neurogenic claudication and sciatica were due to nerve root compression in the lateral recess and/or foraminal stenosis caused by hypertrophied facet joints. In contrast, patients with revision surgery or spine surgery due to bone tumor, spinal fracture, or osteomyelitis, spondylolytic spondylolisthesis, or degenerative scoliosis with Cobb’s angle exceeding 10° on the anteroposterior view were excluded.

### Surgical procedure and postoperative care

All surgeries were carried out by a senior orthopaedic spine surgeon with more than 30 years of experience (C-L. L.) through a posterior midline approach using the Dynesys dynamic stabilization system (Zimmer Biomet Inc, Warsaw, IN, USA). Briefly, paravertebral muscle was dissected bilaterally from the facet joints in the subperiosteal plane, and posterior tension band in the supra/ interspinous ligaments was preserved at the most cephalad facet joint level to avoid adjacent segment degeneration^20^. After removing the spinous process, lumbar stability-preserving decompression with partial bilateral laminectomy and facetectomy were performed at the affected segments as previously described^16^. In patients with severe stenosis, partial facetectomy less than 25% was bilaterally performed for adequate decompression^21^. Then, pedicle screws and the cord and spacer constructs of the Dynesys system were assembled following the manufacturer instructions. Depending on the number of facet joint levels operated on, 2-level or multilevel fixation was performed.

After surgery, all patients were immobilized for two days, and analgesia was prescribed on an as-needed basis. The drainage tube could be removed when the 24-h volume of drainage was less than 100 mL. Patients were required to wear a soft lumbar corset for at least 3 months afterward to support the back and to protect the spine from excessive movement.

### Radiographic evaluation

**Figure 1:**
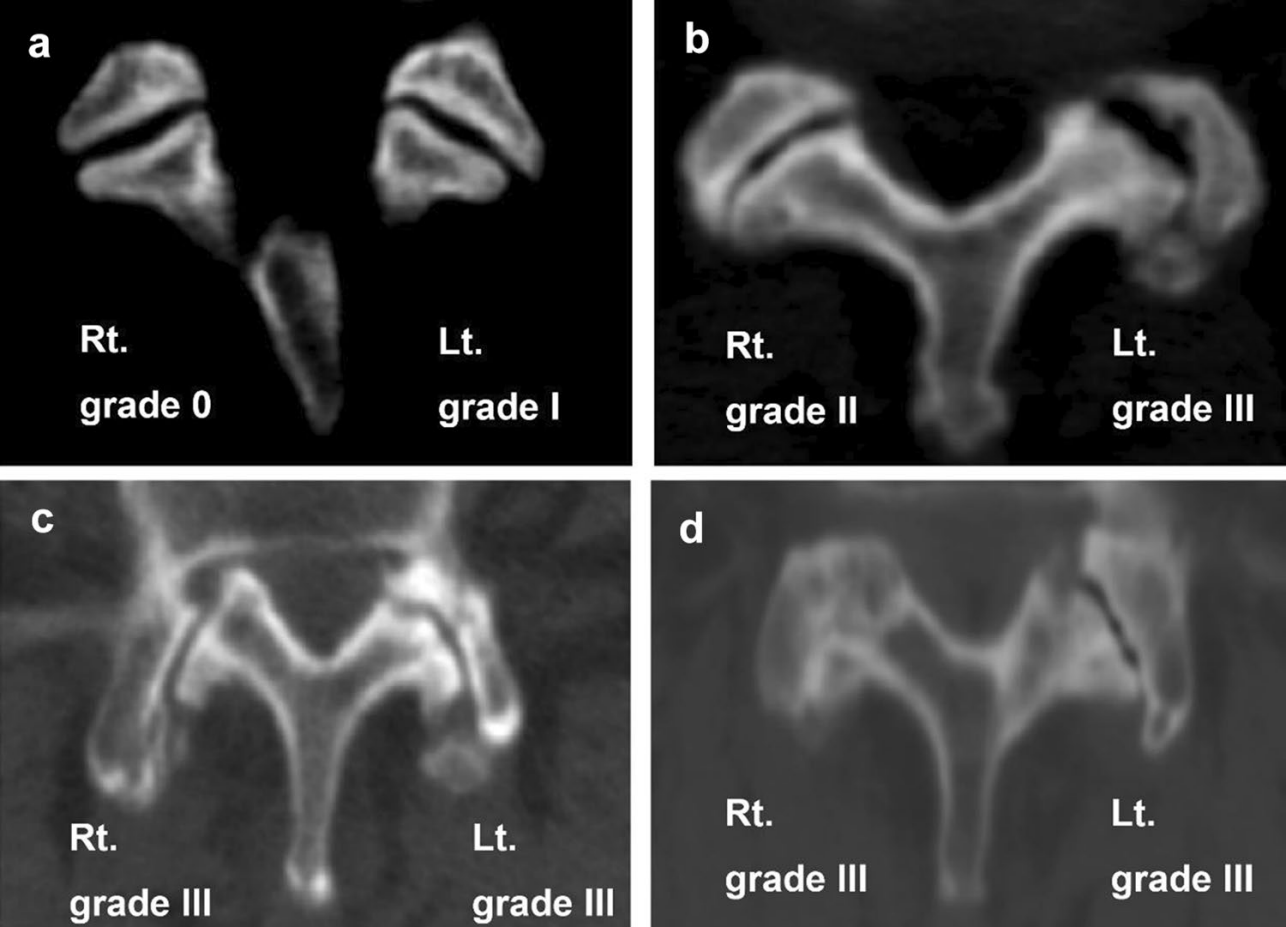
Representative CT images for 4 grades of facet joint arthropathy.

Based on computed tomography (CT) images, facet joint arthropathy of the lumbar spine was graded using a 4-grade scale as proposed by Weishaupt et al.^22^ Weishaupt’s classification of facet joint degeneration has been extensively used^23,24^. Two orthopaedic spine surgeons (P–H. C. and Y-C. Y. with 10-year and 5-years clinical experiences, respectively), who were not involved in the surgical treatment of included patients, graded the severity of facet joint arthropathy independently; disagreements over grading were resolved by consensus. Example CT images for 4 grades of facet joint arthropathy are shown in Fig. 1. We examined 5 facet joint levels, including L1-L2, L2-L3, L3-L4, L4-L5, and L5-S1. Since each level has 2 sides (right and left), each patient had 10 numerical grades of facet joint arthropathy.

In addition, several radiographic spinopelvic parameters were measured preoperatively and postoperatively (at last follow-up visit), such as sacral slope (SS), pelvic tilt (PT), the pelvic incidence (PI), and the L1-S1 lordotic angle that is defined as the angle between L1 superior endplate and S1 superior endplate^25–27^, using the Picture.

Archiving and Communication System (Smart Viewer 3.2; Taiwan Electronic Data Processing Cooperation, Taipei, Taiwan).

### Functional outcome measures

All patients completed two self-reported questionnaires, the VAS and the ODI, before and at last follow-up after surgery; these self-reported measures were then used to evaluate the effect of combined surgical procedures on low back pain. Both instruments have been commonly used for assessments of low back pain in patients undergoing lumber surgery^12–14^. The VAS enables patients to self-rate their back pain on a numerical scale from 0 (none) to 10 (the most severe), and the ODI is a self-completed questionnaire for evaluation of low back pain-related disabilities experienced in daily life.

### Statistical analysis

The numbers of facet joints in each grade and the mean score were statistically compared for each facet joint level and each side of the joints. Categorical variables are presented as numbers and percentages, and were tested by chi-square test or Fisher’s exact test. Continuous variables are presented as mean and standard deviation, and were tested by the Mann–Whitney U test or Kruskal–Wallis test. The demographics and preoperative clinical characteristics were compared between groups using the independent t-test and chi-square test. Subsequently, within-group comparison was conducted to compare the clinical characteristics between before and after surgery in each severity group. Finally, the differences between before and after surgery in clinical characteristics between groups were analyzed using a mixed-effect model adjusted by age. The significance level was set as two-sided (p < 0.05). All statistical analyses were performed using IBM SPSS statistical software version 22 for Windows (IBM Corp, Armonk, NY, USA).

## Results

A total of 88 patients undergoing lumbar-stability-preserving decompression and Dynesys fixation were included in this retrospective study. The preoperative facet joint arthropathy grades of all facet joints are summarized in Table 1, in which the grades of two sides (right and left) for each level were averaged. Regarding the severity of preoperative arthropathy, 5 facet joints (0.6%) were grade 0, 391 (44.4%) were grade I, 287 (32.6%) were grade II, and 197 (22.4%) were grade III. The mean arthropathy score of all facet joints examined was 1.79 ± 0.85. Both the frequency distribution of various grades of facet joint arthropathy and the mean arthropathy scores among distinct facet joint levels were significantly different (both p < 0.001). However, there were no significant differences in the grade distribution and the mean arthropathy scores between the right and left sides of the facet joints (Table 1).

**Table 1:** Grading analysis of preoperative facet joint arthropathy based on Weishaupt’s classification.

Facet joint arthropathy	Grade 0	Grade 1	Grade 2	Grade 3	p value	Mean score	p value
**Total**	5 (0.6)	391 (44.4)	287 (32.6)	197 (22.4)		1.77 ± 0.80	
**Facet joint level**					< 0.001*		< 0.001^§^
L1-2	2 (1.1)	108 (61.4)	57 (32.4)	9 (5.1)		1.41 ± 0.61	
L2-3	1 (0.6)	88 (50.0)	72 (40.9)	15 (8.5)		1.57 ± 0.65	
L3-4	0	65 (36.9)	66 (37.5)	45 (25.6)		1.89 ± 0.78	
L4-5	0	29 (16.5)	46 (26.1)	101 (57.4)		2.41 ± 0.76	
L5-S1	2 (1.1)	101 (57.4)	46 (26.1)	27 (15.3)		1.56 ± 0.76	
**Side**					0.882		0.787
Right	2 (0.5)	196 (44.5)	140 (31.8)	102 (23.2)		1.78 ± 0.80	
Left	3 (0.7)	195 (44.3)	147 (33.4)	95 (21.6)		1.76 ± 0.79	

The data are expressed as number (%) or mean ± standard deviation.

* Statistical differences were examined by Fisher's exact test.

^§^ Statistical differences were examined by Kruskal–Wallis test.

*indicated statistical significance.

The grades of facet joint arthropathy at both sides of operated level(s) for each patient were averaged. The patients with an average grade greater than the mean grade were classified as the more than mean degeneration group (N = 48); the remaining patients were assigned to the less than mean degeneration group (N = 40). The demographics and preoperative clinical characteristics of two facet joint arthropathy groups are presented in Table 2. The mean ages were 57.68 ± 12.49 in the less than mean degeneration group and 60.92 ± 8.37 in the more than mean degeneration group. The majority of included patents did not smoke. In the less than mean degeneration group, 60% of patients had an American Society of Anesthesiology (ASA) I, and 40% had ASA II. In contrast, in the more than mean degeneration group, 47.9% of patients had ASA I, and 52.1% had ASA II. All included patients had grade I spondylolisthesis. The percentages of patients with foraminal stenosis were 40% and 45.8% for the less and more than mean degeneration groups, respectively. Among clinical measurements explored in this study, the mean PT at the more than mean degeneration group was significantly higher than that of the less than mean degeneration group (p = 0.039). The mean PI at the more than mean degeneration group was also significantly higher than that of the less than mean degeneration group (p = 0.008). But, there were no significant differences in the remaining clinical measurements between the two facet joint degeneration groups. The most common comorbidities in the less than mean degeneration group were hypertension (37.5%), Diabetes mellitus (DM, 27.5%), and chronic obstructive pulmonary disease (COPD, 17.5%). In the more than mean degeneration group, the most common comorbidity was also hypertension (35.4%), followed by DM (10.4%) and COPD (10.4%). Finally, the follow-up durations for the less and more than mean degeneration groups were 84.83 ± 27.58 and 92.83 ± 20.45 months, respectively (Table 2).

**Table 2:** Demographics and preoperative clinical characteristics of two facet joint degeneration groups.

Variables	Less than mean degeneration group (n = 40)	More than mean degeneration group (n = 48)	p-value
Sex (Male)	18 (45.0)	12 (25.0)	0.049*
Age	57.68 ± 12.49	60.92 ± 8.37	0.166
Smoking habitat	13 (32.5)	10 (20.8)	0.215
**ASA score**			0.258
I	24 (60.0)	23 (47.9)	
II	16 (40.0)	25 (52.1)	
III	0	0	
**Spondylolisthesis**			NA
I	40 (100.0)	48 (100.0)	
II	0	0	
III	0	0	
**Foraminal stenosis**	16 (40.0)	22 (45.8)	0.582
VAS	6.15 ± 1.37	5.92 ± 1.15	0.386
ODI	52.45 ± 14.76	53.29 ± 15.63	0.797
SS	25.33 ± 11.77	27.57 ± 10.40	0.346
PT	24.06 ± 8.39	27.87 ± 8.57	0.039*
PI	49.40 ± 10.31	55.44 ± 10.58	0.008*
L1-S1 lordotic angle	29.80 ± 13.56	35.26 ± 16.04	0.093
**Comorbidities**			
DM	11 (27.5)	5 (10.4)	0.039*
Hypertension	15 (37.5)	17 (35.4)	0.840
COPD	7 (17.5)	5 (10.4)	0.335
Follow-up duration (months)	84.83 ± 27.58	92.83 ± 20.45	0.133

*VAS* Visual Analogue Scale, *ODI* Oswestry Disability Index, *SS* sacral slope, *PT* pelvic tilt, *PI* pelvic index.

The data are expressed as number (%) or mean ± standard deviation.

Statistical differences were examined by independent t-test or Chi-square test.

*Indicated statistical significance.

Comparison of clinical measurements before and after the combined surgery in two distinct arthropathy groups are shown in Table 3. Postoperative VAS and ODI scores were significantly lower than preoperative values in both less and more than mean degeneration groups (all p < 0.001), indicating significant functional improvement regardless of the preoperative facet joint arthropathy. Postoperative SS values were significantly higher than preoperative values in both facet joint degeneration groups (both p ≤ 0.017), but postoperative PT was significantly lower than preoperative one only in the more than mean degeneration group (p = 0.001). In contrast, PI and L1-S1 lordotic angles were not significantly altered in both facet joint degeneration groups. Moreover, postoperative arthropathy grades of adjacent levels were significantly higher than the corresponding preoperative ones in both facet joint degeneration groups (both p < 0.001; Table 3), indicating continuously worsening after surgery.

**Table 3:** Preoperative and postoperative clinical characteristics stratified by mean degeneration score.

Clinical variables	Less than mean degeneration (n = 40)		p-value	More than mean degeneration group (n = 48)		p-value
	Preoperative	Postoperative		Preoperative	Postoperative	
VAS	6.15 ± 1.37	2.28 ± 2.55	< 0.001*	5.92 ± 1.15	2.08 ± 2.35	< 0.001*
ODI	52.45 ± 14.76	5.50 ± 5.97	< 0.001*	53.29 ± 15.63	5.08 ± 5.47	< 0.001*
SS	25.33 ± 11.77	28.28 ± 10.53	0.017*	27.57 ± 10.40	30.48 ± 10.15	0.007*
PT	24.06 ± 8.39	23.27 ± 8.53	0.536	27.87 ± 8.57	24.36 ± 8.52	0.001*
PI	49.40 ± 10.31	51.54 ± 11.74	0.143	55.44 ± 10.58	54.83 ± 11.28	0.584
L1-S1 lordotic angle	29.80 ± 13.56	29.72 ± 12.29	0.962	35.26 ± 16.04	36.50 ± 13.42	0.411
Grade of adjacent levels	1.51 ± 0.41	1.93 ± 0.61	< 0.001*	1.77 ± 0.61	2.24 ± 0.64	< 0.001*

The data were expressed as mean ± standard deviation.

Statistical differences were examined by paired t-test.

*indicated statistical significance.

**Figure 2:**
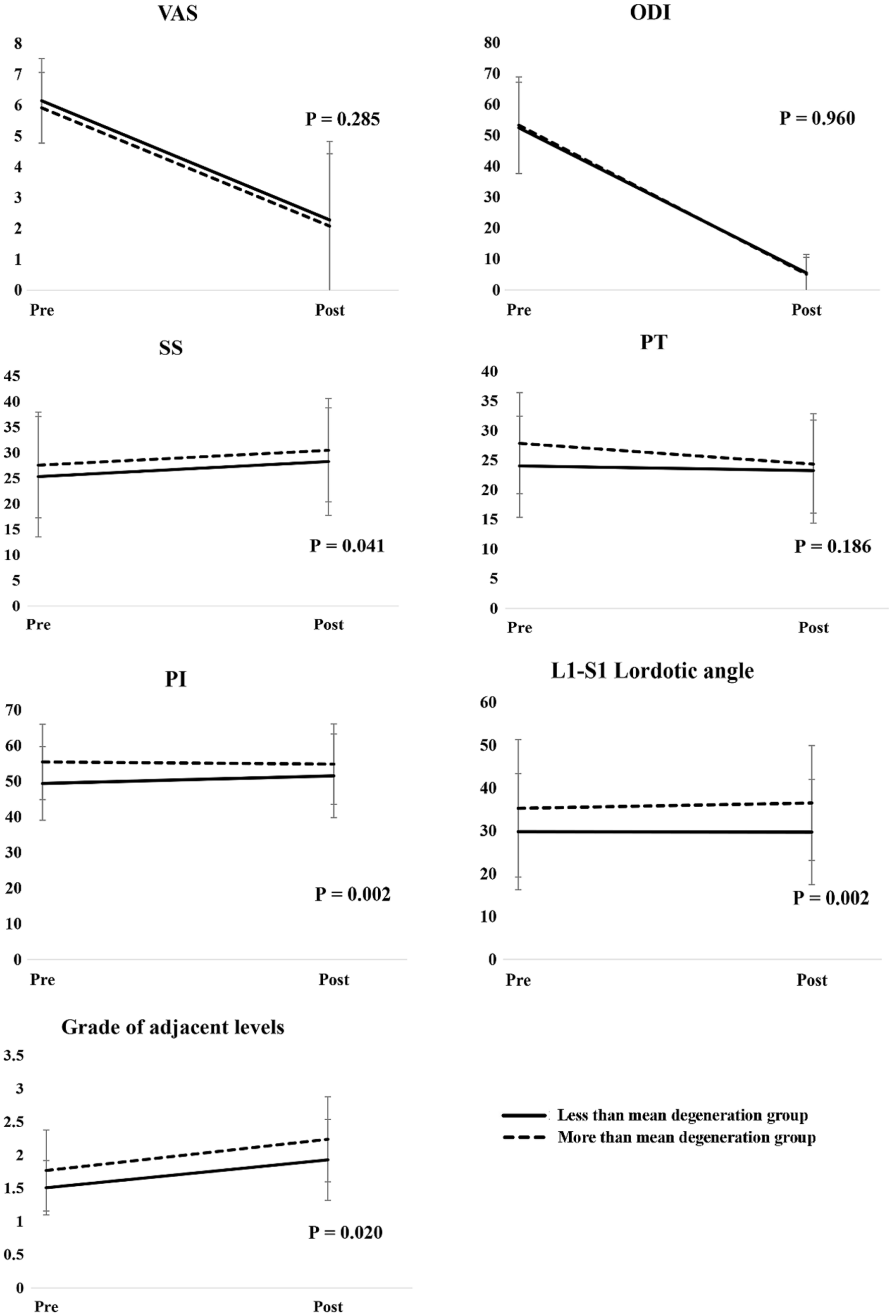
Changes in clinical characteristics before and after operation, stratified by mean preoperative degeneration score. The p-value was calculated based on a mixed-effect model after adjusting for age.

Furthermore, the changes in clinical characteristics from pre-operation to post-operation in two facet joint degeneration groups are shown in Fig. 2. After adjusting for age, there were no significant differences in VAS, ODI, and PT between two facet joint degeneration groups (Fig. 2). In contrast, there were significant between-group differences in SS, PI, L1-S1 lordotic angle, and grade of adjacent level (p = 0.041, p = 0.002, p = 0.002, and p = 0.020, respectively; Fig. 2).

Comparison of surgical complications between two facet joint degeneration groups is shown in Table 4. Rates of incidental dural tear were 10% and 4.2% in the less and more than mean degeneration groups, respectively. Only one patient from the less than mean degeneration group had surgical site infection after surgery. Nine (22.5%) patients with less than mean degeneration and three (6.3%) patients with more than mean degeneration had pedicle screw loosening. When the number of total screws was calculated, incidences of screw loosening were 7.9% and 2.8% for the less and more than mean degeneration groups, respectively. Furthermore, 4 patients (2 less and 2 more) developed symptomatic adjacent segment degeneration (ASD) during follow-up, and revision surgery was subsequently performed. The incidences of various surgical complications were comparable between two facet joint degeneration groups, except for significantly higher screw loosening rate in the less than mean degeneration group (Table 4).

**Table 4:** Comparison of surgical complications between two facet joint degeneration groups.

Surgical complications	Less than mean degeneration group (n = 40)	More than mean degeneration group (n = 48)	p-value
Incidental dural tear	4 (10.0)	2 (4.2)	0.280
Surgical site infection	1 (2.5)	0	0.271
Screw loosening, number of patients (%)	9 (22.5)	3 (6.3)	0.027*
Screw loosening, number of screws (%)	17/216 (7.9%)	6/216 (2.8%)	0.018*
Symptomatic ASD required revision surgery	2 (5.0)	2 (4.2)	1.000

*ASD* adjacent segment degeneration.

The data are presented as number (percentages).

Statistical differences were examined by Chi-square test or fisher exact test.

*indicated statistical significance.

## Discussion

In the present retrospective study, the severity of preoperative lumbar facet joint arthropathy in patients undergoing lumbar-stability-preserving decompression and Dynesys fixation was defined based on the preoperative arthropathy grade of operated levels. The results revealed that the long-term postoperative pain and functional outcomes, as evidenced by the VAS and the ODI scores, were not affected by preoperative facet joint arthropathy severity. The combined surgery significantly increased SS in both the less and more than mean degeneration groups, but only reduced PT in the more than mean degeneration group. And facet joints arthropathy at adjacent levels continued to worsen after surgery in both facet joint degeneration groups. Hence, the preoperative severity of facet joint arthropathy did not affect most long-term clinical outcomes in patients undergoing lumbar-stability-preserving decompression and Dynesys fixation.

The Dynesys dynamic stabilization system utilized pedicle screws to connect spinal motion segments with nonelastic bands, thereby stabilizing the affected lumbar region while allowing some motion of the spine^11,15^. The beneficial effects of the Dynesys dynamic stabilization system on postoperative functional outcomes (the VAS and ODI scores) in patients with lumbar disorders have been previously suggested by a handful of studies with various follow-up durations, including one year^12^, more than 2 years^13^, more than 35 months^14^, from 8 months to 5 years^11^, and from 11.2 to 79.1 months^15^. Furthermore, the combined surgical approach of Dynesys dynamic stabilization and decompression ameliorated low back pain in studies with diverse follow-up periods, such as 9 to 36 months^17^, five years^28^, and more than 10 years^18^. Consistent with the findings of the above-mentioned studies, the present retrospective study with a mean follow-up of 89 months (range: 41 to 131 months) demonstrated that the combination of decompression and Dynesys fixation improved long-term postoperative pain and functional outcomes regardless of preoperative severity of lumbar facet joint arthropathy.

Several radiographic parameters, known as spinopelvic parameters, have been developed to quantify spinopelvic alignment that is critical for maintaining an energy-efficient posture^29,30^. Preoperative measurement of spinopelvic parameters is essential for spine surgeons to propose surgical strategies for correction of sagittal balance^25^. A French retrospective study with a minimum follow-up of 1 year found that the Dynesys dynamic system did not significant alter SS and PT postoperatively^31^. A Korean retrospective study with a minimum follow-up of 4 years reported that SS and PT were not significantly changed after 2-level or multilevel Dynesys dynamic stabilization^32^. However, the present study found that the combination of decompression and Dynesys fixation significantly increased SS regardless of preoperative arthropathy severity. In contrast, the combined surgery reduced PT, but the differences between before and after surgery reached statistical significance only in the more than mean degeneration group. The discrepancy between our findings and others^31,32^ may be in part attributed to distinct spinal disorders, decompression, and/or statistical approaches.

Both TLIF and Dynesys dynamic stabilization have been commonly utilized in the treatment of lumbar facet joint syndrome^12–14^, including multisegmental lumbar degenerative disease^33^. The choice of the surgery type depends mainly on the spine surgeon’s experience and preference, while several issues should be considered for postoperative care. First of all, the Dynesys system might cause stress on the adjacent level above, particularly, after multilevel dynamic stabilization, which should be monitored postoperatively^32^. According to a review of 21 studies^34^, the common complications after Dynesys dynamic stabilization include surgical-site infection (4.3%), pedicle screw loosening (11.7%), pedicle screw fracture (1.6%), and ASD (7.0%). Among 88 included patients in this study, one patient had surgical site infection (1/88; 1.1%), 12 patients had pedicle screw loosening (12/88; 13.6%), and 4 patients developed symptomatic ASD that required revision surgery (4/88; 4.5%). But no pedicle screw fracture was found in this study. It seems that combination of decompression and Dynesys fixation may result in fewer complications, except for pedicle screw loosening. In addition, compared to the conventional fusion technique, non-fusion dynamic stabilization has less risks of developing complications, such as surgical-site infection, cerebrospinal fluid leaks, harvest site pain, and instrumentation failure^35^. However, two studies reported that Dynesys dynamic stabilization and fusion surgery have similar complication rates^33,36^.

Controversial effects of decompression are further complicated the choice of surgical strategy. A randomized controlled trial (RCT) examining pre- and post-operative ODI scores revealed that the long-term efficacy of decompression plus fusion surgery was not better than that of decompression alone in patients with lumbar spinal stenosis^37^. A recent multicenter study also reported that the effect of microdecompression alone in ODI reduction was non-inferior to that of decompression with instrumented fusion in patients with degenerative spondylolisthesis^38^. Both above-mentioned studies suggested decompression alone is a better surgical strategy, although the differences in efficacy between two surgical approaches are not dramatic. However, the clinical superiority of instrumented fusion plus decompression over decompression alone has been suggested by another RCT^39^ and a couple of systematic reviews^40,41^. On the other hand, Schnake et al.^42^ reported that compared with decompression plus fusion, decompression plus dynesys fixation resulted in similar clinical outcomes compared with decompression plus fusion.

A long mean follow-up time of 89.19 ± 24.15 months was the strength of this retrospective study, so long-term postoperative clinical outcomes could be analyzed. On the other hand, this study also had some intrinsic limitations. First of all, only patients undergoing lumbar-stability-preserving decompression and Dynesys fixation were included, and thus there were no control group and/or patients who underwent TLIF fusion surgery for comparison. In addition, this was single-institution study with a small sample size. Hence, further multicenter studies with larger sample sizes are warranted to determine the extent to which preoperative facet joint arthropathy affects spinopelvic alignment after lumbar-stability-preserving decompression and Dynesys fixation.

## Conclusions

The present retrospective study results demonstrated that the preoperative severity of facet joint arthropathy did not alter long-term functional outcomes in patients undergoing lumbar-stability-preserving decompression and Dynesys fixation. However, the extent to which preoperative severity of facet joint arthropathy affects spinopelvic parameters remained to be explored in studies with larger sample sizes. To sum up, the current results suggested that the combination of decompression and Dynesys fixation may be suitable for patients with facet joint arthropathy irrespective of disease severity.

## Data Availability

All data generated or analyzed during this study are included in this article.

## References

[1] Mann SJ, Viswanath O, Singh P (2020). Lumbar Facet Arthropathy.

[2] Zeng ZL, Zhu R, Wu YC, Zuo W, Yu Y, Wang JJ (2017). Effect of graded facetectomy on lumbar biomechanics. J. Healthc. Eng..

[3] Gellhorn AC, Katz JN, Suri P (2013). Osteoarthritis of the spine: The facet joints. Nat. Rev. Rheumatol..

[4] Boody BS, Savage JW (2016). Evaluation and treatment of lumbar facet cysts. J. Am. Acad. Orthop. Surg..

[5] Suri P, Miyakoshi A, Hunter DJ, Jarvik JG, Rainville J, Guermazi A (2011). Does lumbar spinal degeneration begin with the anterior structures? A study of the observed epidemiology in a community-based population. BMC Musculoskelet. Disord..

[6] Goode AP, Marshall SW, Renner JB, Carey TS, Kraus VB, Irwin DE (2012). Lumbar spine radiographic features and demographic, clinical, and radiographic knee, hip, and hand osteoarthritis. Arthritis Care Res. (Hoboken)..

[7] Weinberg DS, Liu RW, Xie KK, Morris WZ, Gebhart JJ, Gordon ZL (2017). Increased and decreased pelvic incidence, sagittal facet joint orientations are associated with lumbar spine osteoarthritis in a large cadaveric collection. Int. Orthop..

[8] McCormick ZL, Choi H, Reddy R, Syed RH, Bhave M, Kendall MC (2019). Randomized prospective trial of cooled versus traditional radiofrequency ablation of the medial branch nerves for the treatment of lumbar facet joint pain. Reg. Anesth. Pain Med..

[9] Choi EJ, Choi YM, Jang EJ, Kim JY, Kim TK, Kim KH (2016). Neural ablation and regeneration in pain practice. Korean J. Pain..

[10] Belykh E, Kalinin AA, Martirosyan NL, Kerimbayev T, Theodore N, Preul MC (2018). Facet joint fixation and anterior, direct lateral, and transforaminal lumbar interbody fusions for treatment of degenerative lumbar disc diseases: Retrospective cohort study of a new minimally invasive technique. World Neurosurg..

[11] Mas Y, Gracia L, Ibarz E, Gabarre S, Pena D, Herrera A (2017). Finite element simulation and clinical follow-up of lumbar spine biomechanics with dynamic fixations. PLoS ONE.

[12] Lee SE, Jahng TA, Kim HJ (2016). Facet joint changes after application of lumbar nonfusion dynamic stabilization. Neurosurg. Focus..

[13] Kuo CH, Chang PY, Wu JC, Chang HK, Fay LY, Tu TH (2016). Dynamic stabilization for L4–5 spondylolisthesis: Comparison with minimally invasive transforaminal lumbar interbody fusion with more than 2 years of follow-up. Neurosurg. Focus..

[14] Tu J, Hua W, Li W, Liu W, Luo R, Li S (2018). Short-term effects of minimally invasive dynamic neutralization system for the treatment of lumbar spinal stenosis: An observational study. Medicine (Baltimore).

[15] Stoll TM, Dubois G, Schwarzenbach O (2002). The dynamic neutralization system for the spine: A multi-center study of a novel non-fusion system. Eur. Spine J..

[16] Louie PK, Paul JC, Markowitz J, Bell JA, Basques BA, Yacob A (2017). Stability-preserving decompression in degenerative versus congenital spinal stenosis: Demographic patterns and patient outcomes. Spine J..

[17] Liu C, Wang L, Tian JW (2014). Early clinical effects of the Dynesys system plus transfacet decompression through the Wiltse approach for the treatment of lumbar degenerative diseases. Med. Sci. Monit..

[18] Veresciagina K, Mehrkens A, Scharen S, Jeanneret B (2018). Minimum ten-year follow-up of spinal stenosis with degenerative spondylolisthesis treated with decompression and dynamic stabilization. J. Spine Surg..

[19] Haher TR, O'Brien M, Dryer JW, Nucci R, Zipnick R, Leone DJ (1994). The role of the lumbar facet joints in spinal stability. Identification of alternative paths of loading. Spine.

[20] Lai PL, Chen LH, Niu CC, Fu TS, Chen WJ (2004). Relation between laminectomy and development of adjacent segment instability after lumbar fusion with pedicle fixation. Spine.

[21] Kiapour A, Ambati D, Hoy RW, Goel VK (2012). Effect of graded facetectomy on biomechanics of Dynesys dynamic stabilization system. Spine.

[22] Weishaupt D, Zanetti M, Boos N, Hodler J (1999). MR imaging and CT in osteoarthritis of the lumbar facet joints. Skeletal Radiol..

[23] Connolly M, Rotstein AH, Roebert J, Grabinski R, Malara F, O'Shea T (2020). Lumbar spine abnormalities and facet joint angles in asymptomatic elite junior tennis players. Sports Med Open..

[24] Winegar BA, Kay MD, Taljanovic M (2020). Magnetic resonance imaging of the spine. Pol. J. Radiol..

[25] Celestre PC, Dimar JR, Glassman SD (2018). Spinopelvic parameters: Lumbar lordosis, pelvic incidence, pelvic tilt, and sacral slope: What does a spine surgeon need to know to plan a lumbar deformity correction?. Neurosurg. Clin. N. Am..

[26] Le Huec JC, Thompson W, Mohsinaly Y, Barrey C, Faundez A (2019). Sagittal balance of the spine. Eur. Spine J..

[27] Iyer S, Sheha E, Fu MC, Varghese J, Cunningham ME, Albert TJ (2018). Sagittal spinal alignment in adult spinal deformity: An overview of current concepts and a critical analysis review. JBJS Rev..

[28] Inose H, Kato T, Yuasa M, Yamada T, Maehara H, Hirai T (2018). Comparison of decompression, decompression plus fusion, and decompression plus stabilization for degenerative spondylolisthesis: A prospective, Randomized Study. Clin. Spine Surg..

[29] Glassman SD, Berven S, Bridwell K, Horton W, Dimar JR (2005). Correlation of radiographic parameters and clinical symptoms in adult scoliosis. Spine.

[30] Lafage V, Schwab F, Patel A, Hawkinson N, Farcy JP (2009). Pelvic tilt and truncal inclination: Two key radiographic parameters in the setting of adults with spinal deformity. Spine.

[31] Chen H, Charles YP, Bogorin I, Steib JP (2011). Influence of 2 different dynamic stabilization systems on sagittal spinopelvic alignment. J. Spinal Disord. Tech..

[32] Kim CH, Chung CK, Jahng TA (2011). Comparisons of outcomes after single or multilevel dynamic stabilization: Effects on adjacent segment. J Spinal Disord Tech..

[33] Wu H, Pang Q, Jiang G (2017). Medium-term effects of Dynesys dynamic stabilization versus posterior lumbar interbody fusion for treatment of multisegmental lumbar degenerative disease. J. Int. Med. Res..

[34] Pham MH, Mehta VA, Patel NN, Jakoi AM, Hsieh PC, Liu JC (2016). Complications associated with the Dynesys dynamic stabilization system: A comprehensive review of the literature. Neurosurg. Focus..

[35] Bonaldi G, Brembilla C, Cianfoni A (2015). Minimally-invasive posterior lumbar stabilization for degenerative low back pain and sciatica. A review. Eur. J. Radiol..

[36] Zhang Y, Shan JL, Liu XM, Li F, Guan K, Sun TS (2016). Comparison of the dynesys dynamic stabilization system and posterior lumbar interbody fusion for lumbar degenerative disease. PLoS ONE.

[37] Forsth P, Olafsson G, Carlsson T, Frost A, Borgstrom F, Fritzell P (2016). A randomized, controlled trial of fusion surgery for lumbar spinal stenosis. N. Engl. J. Med..

[38] Austevoll IM, Gjestad R, Solberg T, Storheim K, Brox JI, Hermansen E (2020). Comparative effectiveness of microdecompression alone vs decompression plus instrumented fusion in lumbar degenerative spondylolisthesis. JAMA Netw. Open..

[39] Ghogawala Z, Dziura J, Butler WE, Dai F, Terrin N, Magge SN (2016). Laminectomy plus fusion versus laminectomy alone for lumbar spondylolisthesis. N. Engl. J. Med..

[40] Liang HF, Liu SH, Chen ZX, Fei QM (2017). Decompression plus fusion versus decompression alone for degenerative lumbar spondylolisthesis: A systematic review and meta-analysis. Eur. Spine J..

[41] Steiger F, Becker HJ, Standaert CJ, Balague F, Vader JP, Porchet F (2014). Surgery in lumbar degenerative spondylolisthesis: indications, outcomes and complications. A systematic review. Eur. Spine J..

[42] Schnake KJ, Schaeren S, Jeanneret B (2006). Dynamic stabilization in addition to decompression for lumbar spinal stenosis with degenerative spondylolisthesis. Spine.

